# Spinal neuroschistosomiasis caused by *Schistoma mansoni*: cases reported in two brothers

**DOI:** 10.1186/s12879-020-05428-2

**Published:** 2020-10-02

**Authors:** Ana Lúcia Coutinho Domingues, Constança Simões Barbosa, Thiago Frederico Andrade Agt, Andréia Braga Mota, Clélia Maria Ribeiro Franco, Edmundo Pessoa Lopes, Rodrigo Loyo, Elainne Christine Souza Gomes

**Affiliations:** 1 Department of Gastroenterology, Clinical Hospital, Federal University of Pernambuco, Av. Prof. Moraes Rego, 1235, Cidade Universitária, Recife, PE 50670-901 Brazil; 2 Department of Parasitology, Schistosomiasis Reference Laboratory, Aggeu Magalhães Institute, Fiocruz - Ministry of Health, Recife, PE 50740-465 Brazil; 3 Department of Neurology, Clinical Hospital, Federal University of Pernambuco, Av. Prof. Moraes Rego, 1235, Cidade Universitária, Recife, PE 50670-901 Brazil

**Keywords:** Spinal neuroschistosomiasis, Schistosomal myeloradiculopathy, *Schistosoma mansoni*, Case report, Diagnosis

## Abstract

**Background:**

Spinal neuroschistosomiasis (SN) is one of the most severe clinical presentations of schistosomiasis infection and an ectopic form of the disease caused by any species of *Schistosoma*. In Brazil, all cases of this clinical manifestation are related to *Schistosoma mansoni*, the only species present in the country. Although many cases have been reported in various endemic areas in Brazil, this is the first time in the literature that SN is described in two brothers.

**Case presentation:**

Two cases of SN were accidentally diagnosed during an epidemiological survey in an urban area endemic for schistosomiasis transmission. Both patients complained of low back pain and muscle weakness in the lower limbs. Sphincter dysfunction and various degrees of paresthesia were also reported. The patients’ disease was classified as hepato-intestinal stage schistosomiasis mansoni at the onset of the chronic form. A positive parasitological stool test for *S. mansoni*, clinical evidence of myeloradicular damage and exclusion of other causes of damage were the basic criteria for diagnosis. After treatment with praziquantel and corticosteroid, the patients presented an improvement in symptoms, although some complaints persisted.

**Conclusions:**

It is important to consider SN when patients come from areas endemic for transmission of schistosomiasis mansoni. Clinical physicians and neurologists should consider this diagnostic hypothesis, because recovery from neurological injuries is directly related to early treatment. As, described here in two brothers, a genetic predisposition may be related to neurological involvement. Primary care physicians should thus try to evaluate family members and close relatives in order to arrive at prompt schistosomiasis diagnosis in asymptomatic individuals and propose treatment in an attempt to avoid progression to SN.

## Background

Schistosomiasis is the parasitic disease that causes the second highest number of deaths around the world, second only to malaria [1]. The same epidemiological pattern is present in Brazil - with schistosomiasis being the parasitosis that causes the greatest number of deaths, after malaria. In Brazil, however, all cases are caused by *Schistosoma mansoni*--the only species of this parasite present in the country. The most recent national parasitological survey estimated that 1.5 million people are infected [2]. Despite the success of the Schistosomiasis Control Program, the severe (hepatosplenic) form of the disease and high mortality continue to pose a major public health problem [3]. Although rare in comparison to the hepatosplenic and intestinal forms of schistosomiasis, another serious manifestation is the ectopic form known as neuroschistosomiasis, which can affect any part of the central nervous system - CNS (brain and spinal cord). Neuroschistosomiasis is the second most common form of presentation of *S. mansoni* infection, beside the hepatosplenic form, although the asymptomatic form of neuroschistosomiasis is far more common than the symptomatic ones. Schistosomal myelopathy--the spinal form of schistosomiasis--tends to occur shortly after infection and is more likely to be symptomatic than cerebral schistosomiasis [4]. Clinical spinal neuroschistosomiasis (SN) syndrome is usually an acute or subacute myelopathy and may or may not be accompanied by polyradiculitis [5]. When *S. mansoni* is involved in the infection, SN with myeloradiculopathy is the most common neurological manifestation [6, 7].

In Brazil, SN is the third most common cause of myelopathy, after trauma and tumors [8]. Incidence of SN is not easy to measure, in view, especially, of the wide range of possible clinical manifestations [9]. However, since 1930, numerous cases of SN have been reported in the literature in Brazil, most of them “case reports” aiming to update the medical and research community regarding the occurrence, clinical presentation, severity and outcome of cases [6, 7, 10–13]. Epidemiologically, one of the most important studies was conducted in the three schistosomiasis mansoni referral hospitals in the Brazilian State of Pernambuco. In 2010, a data survey was carried out in Recife/Pernambuco using medical records covering 15 years of treatment of cases of schistosomiasis. The study gathered cases from departments of neurology and pediatrics, identifying 139 cases of SN in patients aged 2–83 years. This study provided a large quantity of clinical-epidemiological information on SN, including the distribution of the tendon reflex response (Achilles and patellar reflex absent or diminished in 79% of patients), the incidence of spinal cord lesions (40.3% in low thoracic, 15.8% in lumbar; and lowest incidence in lumbar-sacral - 0.7%) and the results of complementary tests: absence of schistosoma eggs in 52% of stool parasitological exams; immunological tests using cerebrospinal fluid (CSF), 70% positive by indirect immunofluorescence and 49.2% by ELISA; Magnetic resonance imaging (MRI) abnormal in 93% of the cases; presence of schistosomal granulomas in rectal biopsy in 55.5% of cases [11].

It is known that the pathophysiology of clinical manifestations of SN involves the presence of *S. mansoni* eggs in the spinal cord causing an inflammatory response in the host, which subsequently results in the formation of a schistosomal granuloma [14, 15], as occurs in other tissues. It is also known that eggs reach the CNS mainly by way of retrograde venous flow through Batson’s venous plexus. The eggs cause a granulomatous reaction strictly proportional to the number of eggs that reach the CNS. This reaction is also associated with the symptoms presented by patients, which include back pain, lower-limb muscle weakness, paresthesia, hypoesthesia, anesthesia, sphincter dysfunction (bladder and intestine), and others [7]. Apart from the wide range of non-specific symptoms, diagnosis of SN is not easy to perform, as there is no single test for the disease. Diagnosis is, therefore, presumptive and based on clinical features and test results, including dysfunctions and/or lesions of thoracic or lumbosacral spinal cord (clinical and image diagnosis), biochemical and immunological analysis of CSF, epidemiological and parasitological confirmation of *S. mansoni* infection, and exclusion of other causes of transverse myelitis [6, 7, 11]. Here we report for the first time in the literature the simultaneous occurrence of SN in two brothers.

## Case presentation

Both cases were identified during a parasitological census survey carried out in Porto de Galinhas, Ipojuca, in the Brazilian State of Pernambuco, between July and December 2019. This location is internationally known not only for its beautiful beaches, but also for having become a new endemic area for schistosomiasis over the past 20 years [15, 16]. This tourist zone is characterized as an urban transmission zone for schistosomiasis, in which foci for transmission of the disease concentrate around artificial breeding sites for vector snails (open sewage ditches) and people are infected in the rainy season when walking in the streets [16, 17]. Most of the 174 confirmed cases of schistosomiasis identified by an actual parasitological survey were residents of the poorest area in the locality, which lacks sanitary infrastructure, as were the two patients in the cases reported here. It is important to note that the vector in this location is the snail of greatest epidemiological importance in Brazil, *Biomphalaria glabrata*.

In both cases, suspicion of SN arose during medical evaluation (November 2019), which included anamnesis and ultrasound. Patients complained of low back pain and difficulty walking. Both were referred to the neurology department of the clinical hospital of the Federal University of Pernambuco for investigation. During hospitalization, tests were performed to investigate other causes of myeloradicular involvement. Both patients presented serum testing negative for the HIV and HTLV retroviruses, negative for syphilis (VDRL), negative for rheumatological problems (antibodies for lupus, Sjogren, APS) and normal for vitamin B12 and copper. The patients also presented normal liver and spleen volume and surface, and absence of ascites in the abdomen ultrasound exam.

Detailed descriptions of each case and its evolution are presented below.

### Case 1

A 36-year-old male artisan, child of non-consanguineous parents, began, in June 2019 (5 months before diagnosis), to experience paresthesia and ascending numbness in the right lower limb, associated with decreased strength in the same limb. The symptoms worsened over the course of several days and the patient reported that, after 2 weeks, the symptoms had spread to the left lower limb with similar characteristics. The patient also stated that, 2 weeks after the onset of the condition, he had experienced difficulty initiating urination, fecal retention and erectile dysfunction. His gait progressively worsened to the point where he was unable to walk without assistance. The patient also reported an intermittent sensation of fever, increased abdominal volume and weight loss of 5 kg. He did not report any strenuous abdominal contraction (Valsalva maneuver) prior to his current complaints. His prior medical history did not reveal any other clinical comorbidities.

A parasitological stool examination using the Kato-Katz method was positive for *S. mansoni,* with 36 eggs per gram of feces (EPG), characterizing a light parasitic load [18]. Ultrasound of the upper abdomen classified the clinical form of the disease as hepato-intestinal, with evidence of DC-pattern periportal fibrosis [19]. Neurological examination revealed the patient to be alert and oriented, but with asymmetric spastic crural paraparesis, predominantly proximal motor deficit, worse on the right, grade 3 muscle power in the left leg and grade 3 minus in the right leg. The patient had normal-active deep reflexes in the upper limbs, higher in the lower limbs, with bilateral cutaneous plantar extensor reflex (Babinski sign) and Achilles clonus. He had well-preserved trophism and tactile hypoesthesia for superficial pain with asymmetric levels of sensation on the right at the sixth dorsal segment (T6) and on the left at the tenth dorsal segment (T10), associated with spastic gait.

**Fig. 1:**
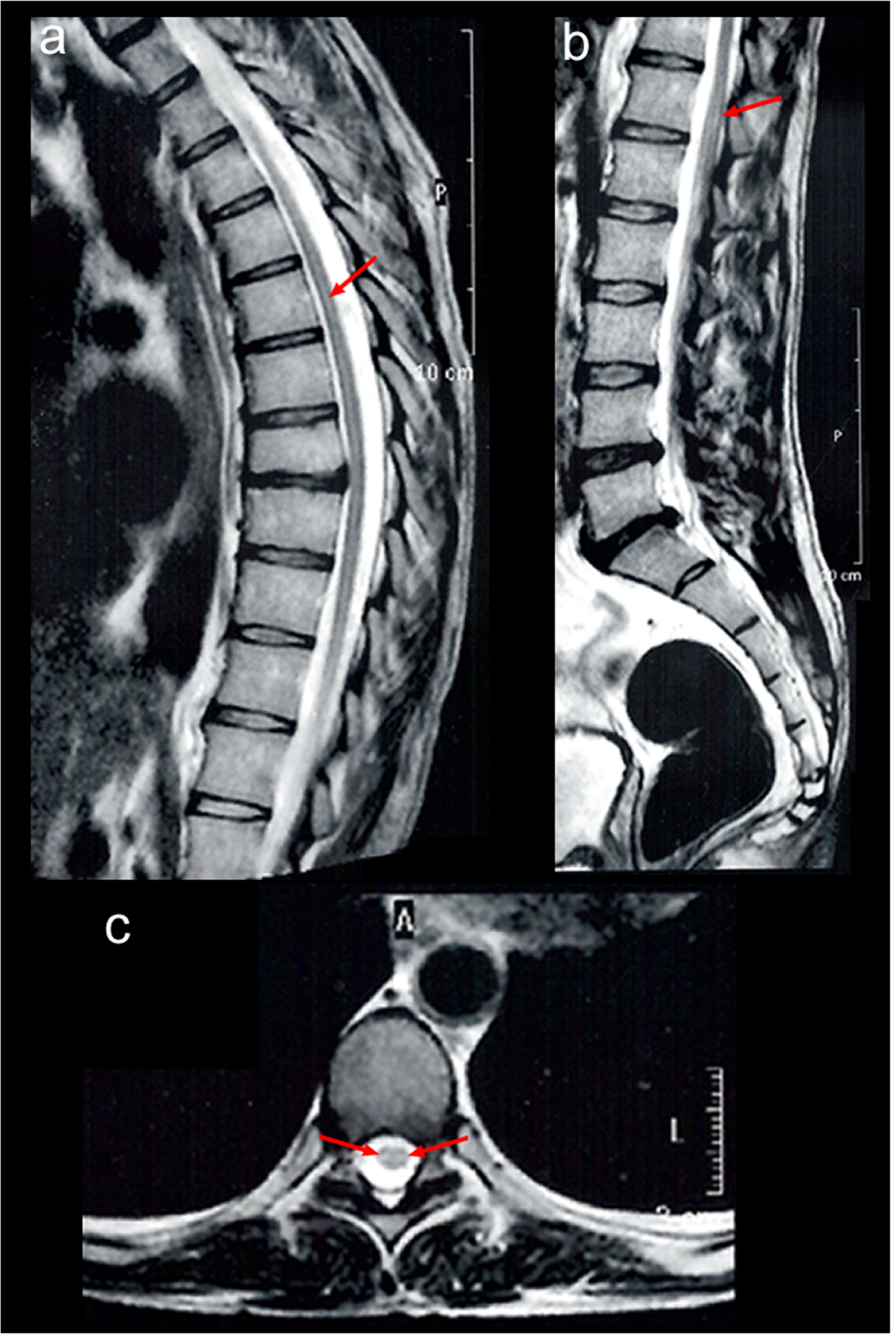
MRI of the thoracic and lumbosacral column of patient 1. **a**/**b**) T2 sagittal sections hyperintensity in thoracic and lumbosacral cord without gadolinium contrast enhancement. **c**) T2 axial sections hyperintensity in thoracic and lumbosacral cord without gadolinium contrast enhancement

The complete blood count (CBS) showed 4650/ mm^3^ leukocytes, with 24% eosinophils. MRI of the spinal cord showed hyperintensity on a T2 sequence along the anterior segment of the thoracic and lumbar spinal cord, without contrast enhancement (Fig. 1). Lumbar cerebrospinal fluid collection showed a normal number of cells and biochemistry, normal values for the ADA test, and a negative VDRL test for syphilis. An aliquot of the CSF was reserved for DNA extraction and investigation of the presence of parasite DNA in the material, using the Loop-mediated isothermal amplification (LAMP) technique, as outlined in a previous publication [20], but insufficient material was collected to be able to perform the examination.

In view of clinical, laboratory and imaging evidence of neuroschistosomiasis, treatment was carried out as recommended by the Brazilian Ministry of Health. This involved high-dose corticosteroid therapy - methylprednisolone 1.0 g IV/day for five consecutive days, a single dose of oral antiparasitic (praziquantel 50 mg/kg), followed by oral corticosteroid therapy (prednisone 1 mg/kg/day) over 6 months [21]. After 15 days, the patient had improved strength - measured on an international scale (modified medical research council) [22, 23]—from grade 3 to 4 in the left leg and from grade 3 minus to 4 in the right leg. This represented a significant improvement in sensitivity, although sphincter complaints and spasticity persisted. Use of oral corticosteroid therapy was continued for 6 months and muscle relaxants were prescribed for spasticity.

### Case 2

A 37-year-old male patient, a general services assistant, child of the same parents as Case 1, began to experience low back pain radiating to the posterior thigh, similar to the sensation of being burnt or pricked by needles. This was followed by a decrease in tactile-pain sensation in the same region for more than 4 months prior to diagnosis. He did not report any abdominal contraction effort (Valsalva maneuver) prior to his current complaints. Two weeks after the onset of symptoms, the patient developed symmetrical weakness in the lower limbs and difficulty getting up from a sitting position. He also complained of sphincter problems, such as sporadic urinary incontinence around twice a week. These symptoms had progressed over the previous 2 months, mainly with pain in the right leg. No other clinical comorbidities appeared in the patient’s medical history.

The Kato-Katz parasitological stool examination was positive for *S. mansoni* and identified 144 EPG, characterizing a moderate parasitic burden [18]. Ultrasound of the upper abdomen classified the clinical form of the disease as hepato-intestinal, with evidence of C-pattern periportal liver fibrosis [19]. Neurological examination showed the patient to be alert and oriented, but with predominantly proximal asymmetric spastic crural paraparesis (grade 3 in the left leg and grade 4 in the right leg), worse on the left. The patient had well-preserved trophism and presented bilateral pain-tactile hypoesthesia in the lumbosacral roots (L4-L5 and L5-S1) and pareto-spastic gait, worse in the left leg.

**Fig. 2:**
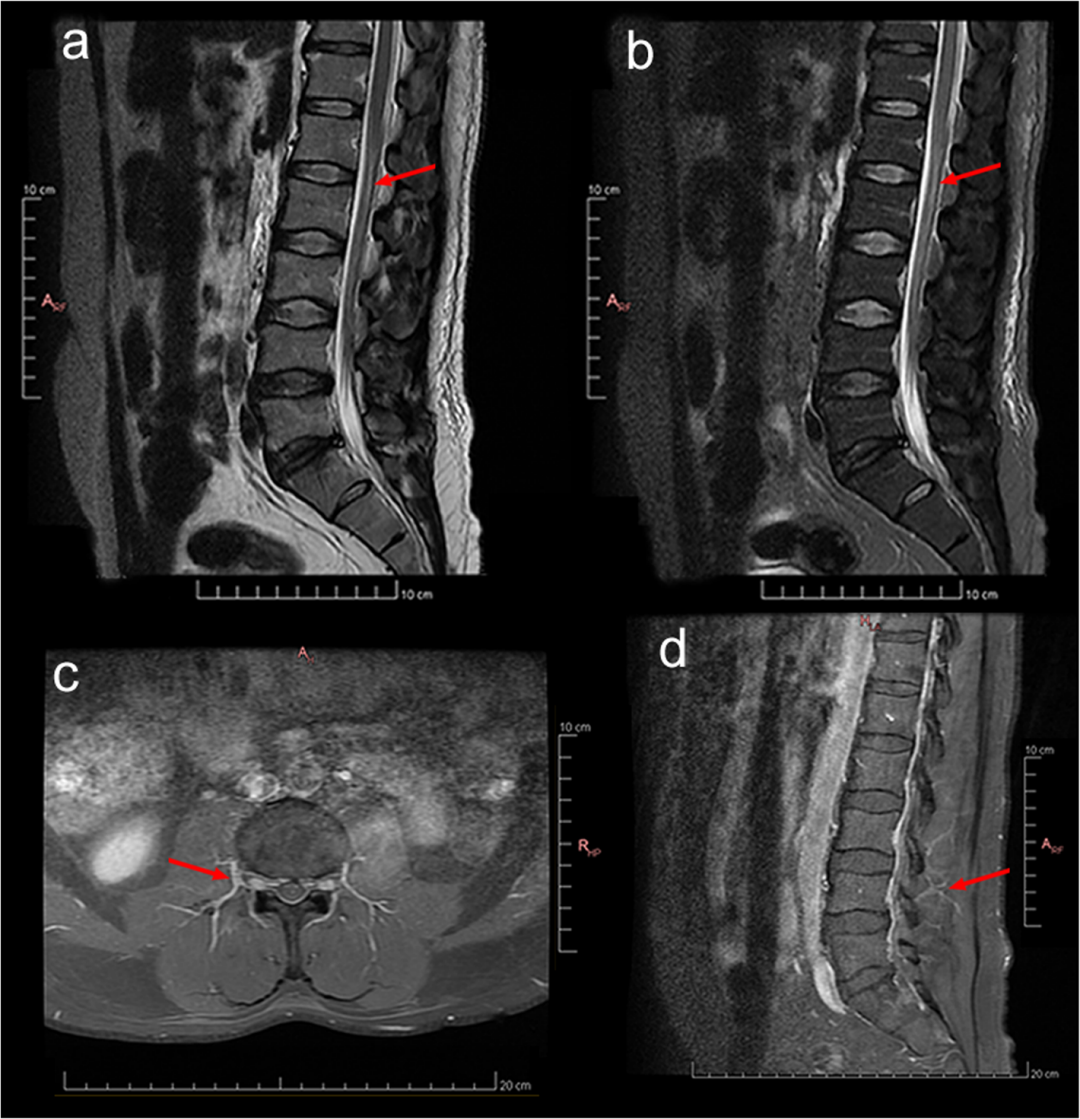
MRI of the lumbosacral column of patient 2. **a** Sagittal T2 with root hypersignal at the cauda equina level. **b** Sagittal T2 with fat suppression with hypersignal at the cauda equina level. **c** Axial T1 with gadolinium contrast with root thickening at the L4 vertebral level and slight gadolinium contrast enhancement. **d** Sagittal T1 section with root thickening at the L4 vertebral level and slight gadolinium contrast enhancement

The CBC showed a total of 7400/ mm^3^ leukocytes, with 8% eosinophils. MRI of the spinal cord showed hyperintensity in a T2 sequence along the anterior segment of the lumbar spinal cord (Fig. 2 a/b), with thickening of the lumbosacral spinal roots, using gadolinium contrast to study the T1 sequence (Fig. 2 c/d). Thoracic segment showed no abnormalities. Lumbar cerebrospinal fluid collection revealed normal cell numbers and biochemistry and normal values for the ADA test. The VDRL test was negative for syphilis. An aliquot of the CSF was reserved for DNA extraction and investigation of the presence of parasite DNA in the material, using the loop-mediated isothermal amplification (LAMP) technique. The test was positive for the presence of *S. mansoni* DNA in CSF.

This patient received the same oral corticosteroid and antiparasitic therapy as Case 1. After 15 days, the patient had improved strength - measured on an international scale (modified medical research council) [22, 23]—from grade 3 to 4 in the left leg and grade 4 to 5 in the right leg, with no sphincter issues. Bilateral pain-tactile hypoesthesia in the lumbosacral roots, however, persisted in this patient (L4-L5 and L5-S1). Oral corticosteroid therapy was continued for 6 months, along with muscle relaxants to treat spasticity. Pregabalin has been used as a first line agent for the treatment of central neuropathic pain and was thus used to treat neuropathic pain in this patient [24].

Both patients will continue to be followed up in the neurology and gastroenterology department until such a time as the clinical sequelae improve.

## Discussion and conclusions

Although SN can affect both men and women in the same way, both cases presented here occurred in young adult males. These results corroborate a number of studies reporting SN in children and young adults, most of them male [10, 11, 25]. These findings can be partially explained by the fact that males and young people experience greater exposure to situations associated with schistosomiasis infection, such as entering fresh water containing snail vectors. In the particular case of urban transmission, young males are most likely to move through these localities during times of flooding, which are the periods when the disease is most highly transmissible [26]. It is important to note that the two cases described here share the same behavior related to risk of *Schistosoma* infection. Both brothers live in the neighborhood in the region that is most at risk for schistosomiasis infection, since it is the poorest in the locality and contains the largest number of breeding sites for *Biomphalaria glabrata* (14/36) and foci of transmission of the disease-- breeding sites with snails releasing cercaria (7/9).

Although physical effort involving heightened abdominal pressure has been associated with the occurrence of SN, none of the patients reported heavy work that could facilitate the passage of eggs through the Batson plexus [7, 13]. However, the fact that these two brothers both developed SN may indicate that they present anastomosis of the veins of the portal-mesenteric and cerebrospinal system [27–29]. Another important finding that may help to explain the occurrence of SN in these two brothers is that, of the 174 cases identified with Kato-Katz positive among the 2193 individuals who participated in the parasitological survey in the locality covered, these two cases were the only ones that presented SN. In view of these data, it is worth considering the possibility of some as yet unidentified genetic predisposition being responsible for the development of the neurological form in both brothers with schistosomiasis mansoni.

This is the first report of SN in siblings but, in France, similar cases have been reported in two brothers returning from a vacation in Madagascar. Both brothers had a history of bathing in stagnant freshwater until intense generalized pruritus forced them to leave the water. Schistosomiasis infection was confirmed by a stool examination testing positive for *S. mansoni* and cerebral neuroschistosomiasis was also confirmed [30]. Both cases reported here were in the initial chronic stage of the disease, diagnosed by ultrasound and blood count as the hepato-intestinal form of schistosomiasis, indicating that patients could have been infected in the previous few months and were in the phase of established active infection, with inflammatory response against parasite eggs [19, 31]. The immunological response has been implicated in the clinical manifestation of SN. Some studies have indicated that CNS lesions and SN symptoms are more accentuated among recently infected individuals [25, 30], when the patient is evolving from the acute to the chronic phase, presenting the hepato-intestinal form of the disease [7], as occurred in the two cases reported here. It is important to note that Patient 1 had more symptoms and greater neurological impairment, even though he presented the lower parasite load and greater damage to the liver (fibrosis), compatible with a stronger immune response.

All the symptoms presented in these cases have been extensively described in the literature and are expected manifestations of SN. Low back pain, lower-limb muscle weakness, some kind of lower-limb paresthesia (symmetric or asymmetric) and gait disorder have been described in most cases of SN [6, 7, 10, 11, 14, 15, 32]. A prospective study of 63 SN patients found bladder, intestinal and sexual dysfunction in 98.6, 87.0 and 91.4%, respectively [7, 32], in keeping with the symptoms presented by the patients studied here. A diagnosis of neuroschistosomiasis should be considered whenever a patient with a history of exposure to schistosome-infected water presents either seizure, ataxia, increased intracranial pressure, hemianopsia, nystagmus and vertigo or paraplegia, sphincter dysfunction, or sensory disturbances from the pelvic girdle down, as these are the neurological alterations usually produced by cerebral or spinal cord SN [13].

MRIs of the spinal cord have shown enhancement of the medullar signal. A fine, diffuse, nodular heterogeneous pattern of contrast enhancement after gadolinium administration has been described. Both of these are changes compatible with inflammation and/or demyelination in the thoraco-lumbar segment and lumbosacral roots. The lower thoracic to lumbosacral segments of the spine represent the main area damaged by *S. mansoni* eggs in cases of SN [7, 11]. MRI is considered more sensitive than computer tomography but both methods may show evidence of spinal cord atrophy in the later phase of the disease [13, 33].

Other complementary tests, such as serology for other infectious diseases associated with symptoms of myelitis, were performed to rule out other causes of this manifestation, as recommended by the Centers for Disease Control and Prevention [34]. CSF very reliably reflects the inflammatory process that damages the spinal cord, which includes lymphomononuclear hypercellularity associated with the presence of eosinophils, an increase in the concentration of proteins, and the presence of antibodies for *S. mansoni*, although it is possible to find cases with a normal CSF profile [4, 13]. The use of LAMP is an especially promising auxiliary method for diagnosis of SN, as it is an extremely sensitive and specific method for detecting the presence of *S. mansoni* DNA in biological samples [31]. LAMP has been proved to be efficient by one parasitological survey, presenting sensitivity of 92.86% and specificity of 80.11% in stool samples [20], which are much more difficult to process. It is important to note that another type of molecular test similar to LAMP, real-time PCR, has already been used to diagnose SN in CSF samples [35].

After treatment, which followed the national (Brazilian Ministry of Health) and international recommendations by administrating corticosteroids to reduce the inflammatory process and clinical manifestations and treating schistosomiasis with praziquantel [15, 21], the two patients presented some improvement in symptoms. Case 1, however, continued to experience urinary and fecal retention and spasticity of gait and Case 2 continued to exhibit sensitivity and worsening back pain. As reported in the literature, the outcome of SN is directly related to early diagnosis and treatment [7]. In the cases reported here, the patients were diagnosed 4 and 5 months after the onset of symptoms, in the early chronic phase of the disease and this may be directly related to their poor response to treatment. Both were therefore put on a six-month regimen of corticosteroid therapy and prescribed chronic pain medications, such as anticonvulsants and muscle relaxants. A second *S. mansoni* treatment may be necessary to ensure all worms were killed.

Finally, the publication of these cases of neuroschistosomiasis caused by *S. mansoni*, involving two brothers, should alert the primary care physicians to the need for early diagnosis of this form of presentation of infection in endemic areas. Furthermore, after diagnosis of a case, screening for other cases in the region should be undertaken, especially in family members and close relatives, since a genetic predisposition may be related to neurological involvement. Early diagnosis may thus help to ensure prompt treatment and improve the prognosis for neuroschistosomiasis.

## Data Availability

All data and materials are available with the corresponding author.

## Abbreviations

ADA: Adenosine deaminase
APS: Antiphospholipid syndrome
CBS: Complete blood count
CNS: Central nervous system
CSF: Cerebrospinal fluid
DNA: Deoxyribonucleic acid
EPG: Eggs per gram of feces
HIV: Human immunodeficiency virus
HTLV: Human T lymphotropic virus
IV: Intravenous
KG: Kilogram
LAMP: Loop-mediated isothermal amplification
MG: Milligram
MRI: Magnetic resonance imaging
*S. mansoni*: *Schistosoma mansoni*
SN: Spinal neuroschistosomiasis
VDRL: Venereal Disease Research Laboratory
